# Machine learning applied to epilepsy: bibliometric and visual analysis from 2004 to 2023

**DOI:** 10.3389/fneur.2024.1374443

**Published:** 2024-04-02

**Authors:** Qing Huo, Xu Luo, Zu-Cai Xu, Xiao-Yan Yang

**Affiliations:** ^1^School of Nursing, Zunyi Medical University, Zunyi, China; ^2^School of Medical Information Engineering, Zunyi Medical University, Zunyi, China; ^3^Department of Neurology, The Affiliated Hospital of Zunyi Medical University, Zunyi, China

**Keywords:** machine learning, epilepsy, VOSviewer, CiteSpace, visual analysis

## Abstract

**Background:**

Epilepsy is one of the most common serious chronic neurological disorders, which can have a serious negative impact on individuals, families and society, and even death. With the increasing application of machine learning techniques in medicine in recent years, the integration of machine learning with epilepsy has received close attention, and machine learning has the potential to provide reliable and optimal performance for clinical diagnosis, prediction, and precision medicine in epilepsy through the use of various types of mathematical algorithms, and promises to make better parallel advances. However, no bibliometric assessment has been conducted to evaluate the scientific progress in this area. Therefore, this study aims to visually analyze the trend of the current state of research related to the application of machine learning in epilepsy through bibliometrics and visualization.

**Methods:**

Relevant articles and reviews were searched for 2004–2023 using Web of Science Core Collection database, and bibliometric analyses and visualizations were performed in VOSviewer, CiteSpace, and Bibliometrix (R-Tool of R-Studio).

**Results:**

A total of 1,284 papers related to machine learning in epilepsy were retrieved from the Wo SCC database. The number of papers shows an increasing trend year by year. These papers were mainly from 1,957 organizations in 87 countries/regions, with the majority from the United States and China. The journal with the highest number of published papers is EPILEPSIA. Acharya, U. Rajendra (Ngee Ann Polytechnic, Singapore) is the authoritative author in the field and his paper “Deep Convolutional Neural Networks for Automated Detection and Diagnosis of Epileptic Seizures Using EEG Signals” was the most cited. Literature and keyword analysis shows that seizure prediction, epilepsy management and epilepsy neuroimaging are current research hotspots and developments.

**Conclusions:**

This study is the first to use bibliometric methods to visualize and analyze research in areas related to the application of machine learning in epilepsy, revealing research trends and frontiers in the field. This information will provide a useful reference for epilepsy researchers focusing on machine learning.

## 1 Introduction

Epilepsy is a common, chronic, disabling neurological disorder that affects ~50 million people of all ages worldwide (1, 2). The World Health Organization has recognized epilepsy as a public health priority, and about a quarter of all seizures are preventable (3, 4). Over the past two decades, research on the use of machine learning (ML) in epilepsy has grown exponentially, informing the accurate identification and prevention of seizures and potentially reshaping our approach to clinical diagnosis, prediction of therapeutic outcomes, and management of cognitive comorbidities in epilepsy (5, 6).

ML is a subfield of artificial intelligence (AI), which is a scientific discipline that focuses on how computers learn from data (7, 8). ML, a data-driven technology, turns raw data into actionable and interpretable insights that support clinical decision-making (9). It tackles the challenge of developing computer models that improve autonomously with experience. Positioned at the crossroads of computer science and statistics, ML is pivotal in AI and data science, experiencing rapid growth (10). Beyond its extensive application in image recognition, language processing, and data mining, ML's adoption in healthcare is noteworthy (11). It is leveraged for a range of purposes, including automated imaging analysis and disease prediction. In the specific context of epilepsy, ML techniques are employed for detecting and testing seizures, predicting epilepsy in at-risk individuals, classifying types of epilepsy, modeling the disorder, tracking and forecasting responses to pharmacological and surgical interventions, analyzing EEG data, and enhancing neuroanatomical localization and bias regularization (5, 6, 9, 11–13). Consequently, ML plays an essential role in advancing epilepsy research.

Notably, more and more articles on ML and epilepsy are published every year. Therefore, it is important for researchers to keep a constant and sizable update on the latest literature. Bibliometric analysis is a method of qualitative and quantitative analysis of research in a specific field of study over a specific period of time using mathematical and statistical methods (14). It not only provides a basis for researchers to analyze the hotspots and development trends of the discipline and predict the direction of development of the discipline, but also provides a reference for the construction of the discipline and training of talents in the hospital. It further provides active research institutions and groups that can facilitate potential international or national research collaborations (15–18). Despite the rapid development of research related to the application of ML in epilepsy, there is still a lack of bibliometric analysis related to this field. Therefore, the main research objective of this study is to conduct a bibliometric and visual analysis of ML applications in epilepsy over the past two decades, with the aim of providing new insights for future research in this field.

## 2 Materials and methods

### 2.1 Data sources

**Figure 1:**
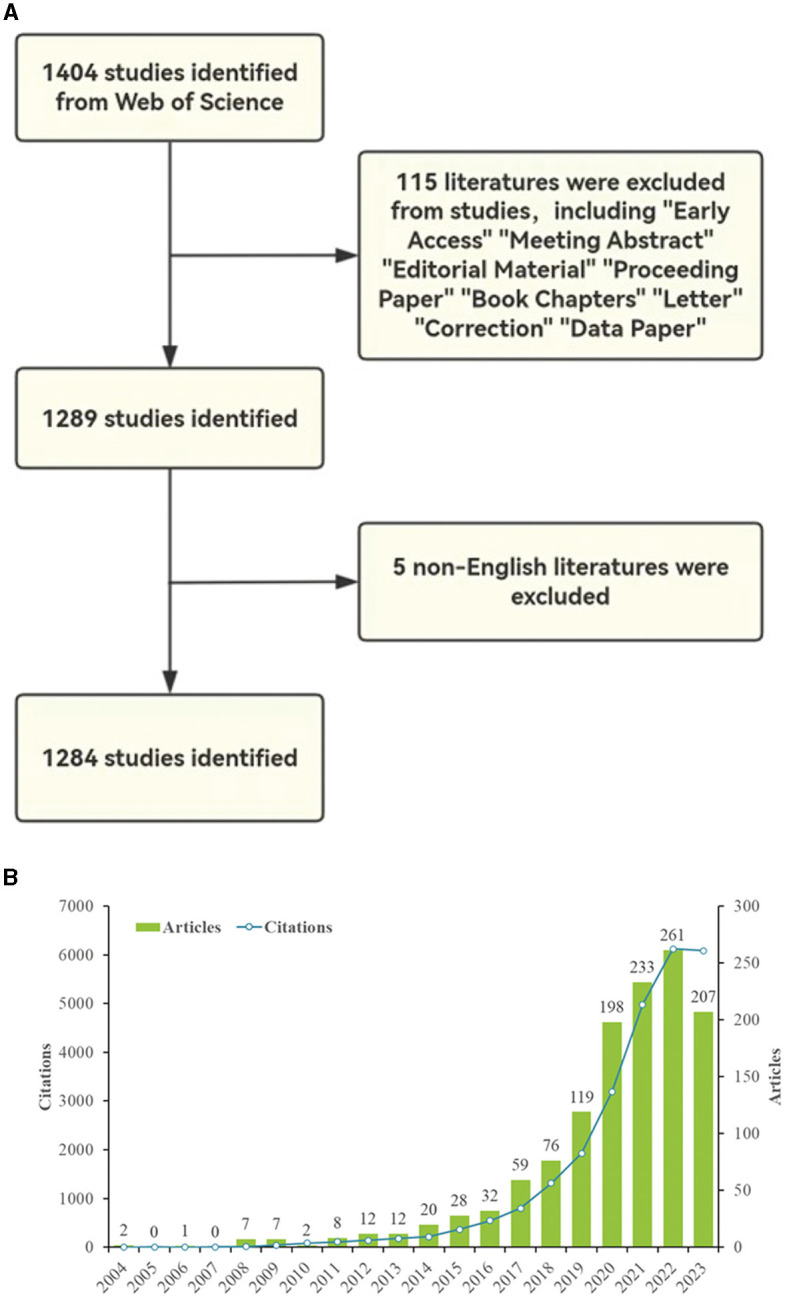
**(A)** Flowchart and annual results of paper screening for ML in epilepsy research, 2004–2023. **(B)** Annual publication volume and annual citation frequency of relevant articles in the last two decades (2023 data as of December 22nd).

The bibliometric analysis data for this study were obtained from the Web of Science Core Collection (WOSCC), a comprehensive standardized database widely used in bibliometric studies (3). In WOSCC, TS stands for Topic Sentence. The search formulas used in this study were set to TS = (“machine learning” OR “Learning Machine” OR “Transfer Learning” OR “Learning Transfer”) and TS= (“epilep^*^”). The search period was limited to January 1, 2004 to December 22, 2023. Only “article” and “review article” were selected as article types, and the language was limited to English, resulting in 1,284 articles (Figure 1A). Based on the above formula, the results were exported to plain text files in txt and csv formats for searching on WOSCC. The search was completed on December 22, 2023 to prevent data bias due to database updates.

### 2.2 Eligibility criteria

The study applied specific inclusion and exclusion criteria. Inclusion criteria included investigations exploring the application of ML in epilepsy, including original research articles and reviews in English-language publications. Conversely, exclusion criteria included early access, meeting abstract, editorial material, proceeding paper, book chapters, letter, correction and data paper. Discrepancies were resolved through discussion and, if needed, adjudicated by a third researcher (XXY). If consensus could not be reached, the final decision was made by a senior neurologist (ZCX).

### 2.3 Data analysis and visualization

CiteSpace, developed by Chao-mei Chen, is the widest used bibliometric analysis software (19), CiteSpace by focusing on identifying and visualizing trends, bursts, and pivotal points in scientific literature. It is particularly adept at uncovering emerging trends and transitions in research topics, as well as highlighting influential studies. We used CiteSpace 6.2.R6 Advanced and VOSviewer visualization software to analyze country and region distribution and collaboration, institutional collaboration and publication volume, author distribution and collaboration, dual graph overlay of journals and keyword bursts, and reference collaboration. VOSviewer, developed by Leiden University in the Netherlands, is mainly used for bibliometric network graph analysis (20), VOSviewer is chosen for its robust capability in visualizing complex networks of co-authorship, co-citation, and keywords within scientific literature. It excels at mapping and clustering large sets of data, making it invaluable for identifying the main research areas, key authors, and institutions driving epilepsy research. We analyzed dual graph overlay of journals, keyword bursts, and reference clustering used CiteSpace 6.2.R6 advanced visualization software. Clustering was performed automatically based on the similarity matrix and VOS mapping techniques, and then appropriate labels were added to differentiate based on content. In addition, we visualized references and keywords using R software (version 4.3.2, using the Bibliometrix R package and its tool Biblioshiny) (http://www.bibliometrix.org) (21), and Microsoft Excel 2019 to present the publication and citation trends of the literature over the years. Bibliometrix excels in statistical analysis, enabling researchers to dissect the data further and perform comparative analyses, trend analyses, and thematic explorations. This tool is particularly useful for extracting detailed insights from the data, supporting evidence-based conclusions about the state and progress of research in epilepsy. All raw data used in this study were obtained from Web of Science public databases and therefore no ethical review was required.

## 3 Result

### 3.1 Global overview

Based on the search strategy, 1,284 articles (1,162 articles and 122 reviews) were screened from the WOSCC database from 2004 to 2023. The average number of citations per article was 20.14, and as a relatively new research area, nine of them had more than 50 citations. Overall, 550 authors from 87 regions and countries published relevant literature on ML applications in epilepsy in 749 journals worldwide.

### 3.2 Annual publications and citation trends

Figure 1B shows the annual number of publications and annual citation frequency of relevant articles over the last two decades (2004–2023). As a whole, there is a general upward trend in the number of annual papers related to the application of ML in epilepsy. In the first decade the field is still in the initial exploration stage, so the number of papers and citation frequency are low. However, the latter decade has seen an explosive growth, which is related to the wide application of AI in medicine. The literature in this field also shows an overall upward trend in terms of annual citation frequency, and the upward trend in citation frequency is more obvious after 2019. In 2022, the number of annual publications and annual citation frequency reached an all-time high of 261 articles and 6,124 citations, respectively. The decline in 2023 may be due to the fact that some articles have not yet been published.

### 3.4 Distributions of countries/regions

Currently, 87 countries/regions are involved in research on the application of ML in epilepsy. The top 10 countries accounted for 70% of the combined publication output in this field (Table 1). The United States has the highest number of publications (279 articles), citations (7,470) and co-publications (multinational publications: 81) in this discipline. China and India have the second and third highest number of publications. The United States and China are the main forces behind research on ML applications in epilepsy. Although the number of publications in China is comparable to that of the United States (279 in the United States, 268 in China), there is a huge gap in the citation frequency between the two countries (7,470 in the United States, 4,126 in China), indicating that the United States has a much higher academic reputation than China in this research field. Overall, ML research in epilepsy is more limited, mostly with national collaborations, and the lack of international collaborations could limit the development of the field, which has to be addressed in the future.

**Table 1:** The 10 most productive countries in terms of research on ML applications in epilepsy.

**Rank**	**Country**	**Articles**	**Citations**	**SCP**	**MCP**	**MCP ratio MCP**
1	USA	279	7,470	198	81	0.29
2	China	268	4,126	214	54	0.201
3	India	116	2,178	89	27	0.233
4	Australia	54	1,376	30	24	0.444
5	Canada	48	1,136	23	25	0.521
6	United Kingdom	47	1,089	21	26	0.553
7	Germany	40	858	22	18	0.45
8	Turkey	31	1,057	30	1	0.032
9	Italy	30	590	21	9	0.3
10	Saudi Arabia	26	785	13	13	0.5

SCP, single country publications; MCP, multiple country publications.

**Figure 2:**
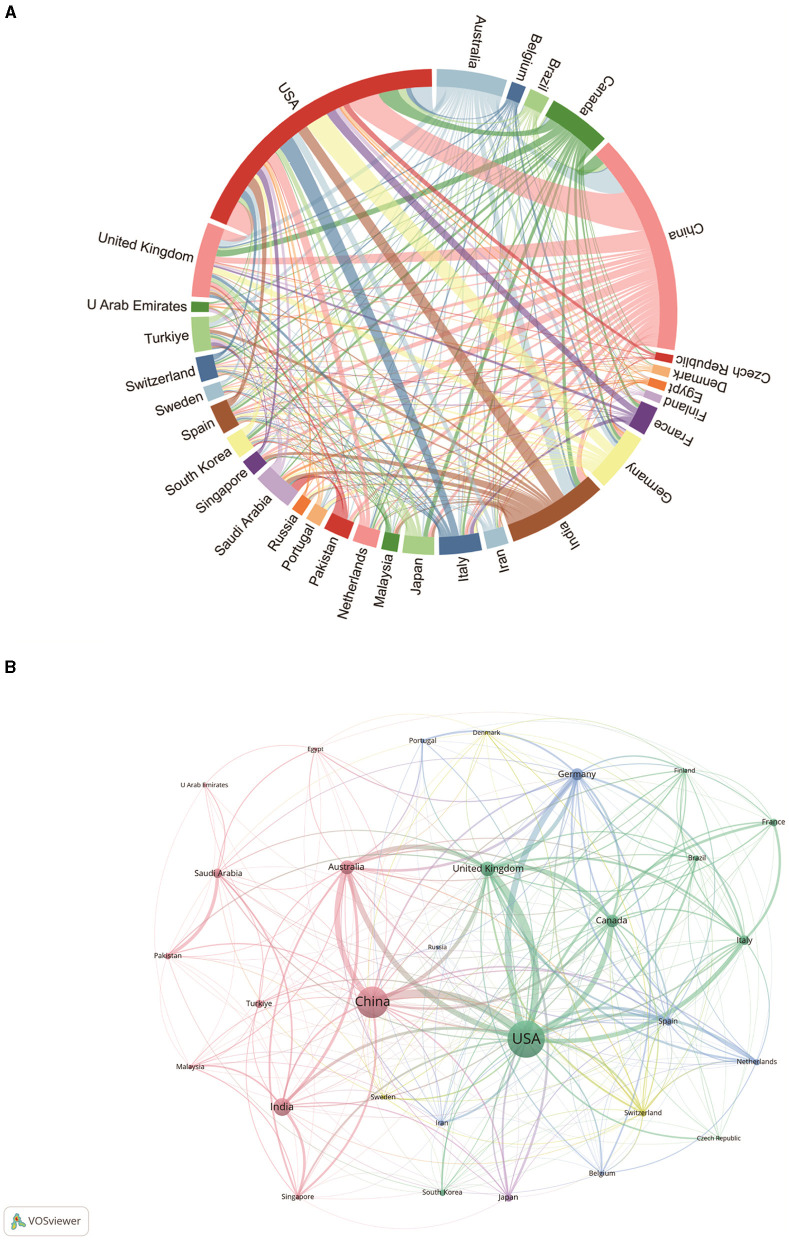
ML and epilepsy related countries/regions analysis. **(A)** Chord plot of country/region cooperation. Each external curve represents a country/region, and the thickness of the line is directly related to the strength of cooperation between countries/regions. **(B)** Visualization of the cooperation network between countries/regions using VOSviewer. The figure shows the top 30 countries/regions in terms of the number of documents. Nodes of different colors represent different clusters of countries/regions, and the size of the nodes corresponds to their respective saliency.

Next, we plotted the top 30 countries/regions in terms of the number of publications in this area on a country cooperation map (Figures 2A, B). Each segment of the curve at the periphery of the chord chart in Figure 2A corresponds to a particular country or region, and the length of the segment represents the number of publications in that region. In addition, the thickness of the connections between countries reflects the closeness of cooperation. The longest peripheral curves and the strongest line between China and the United States indicate that the two countries have the highest number of paper outputs, and the frequency of academic collaborations between the two countries exceeds that of any other pair of countries (Figure 2B), which may be attributed to the fact that in recent years, the global network of epilepsy researchers has continued to develop and grow, which provides a platform for scholars in China and the United States to carry out collaborative research. The number of paper outputs often reflects the status of a country or region in the discipline (22). China and the United States have the highest number of publications in the field of “ML in epilepsy,” which demonstrates the academic status and disciplinary influence of both countries. The close cooperation between these two countries is expected to promote future disciplines in this field (6), such as improving the accuracy of predicting epileptic seizures.

Figure 2B shows the collaboration between countries involved in research on the application of ML in epilepsy with the top 30 publications. In VOSViewer, countries/regions are divided into five main clusters based on the closeness of collaboration, indicated by different colors. The green clusters mainly include countries such as the United States, the United Kingdom, Canada, Italy, and France; the red blocks mainly include countries such as China, Australia, the United Kingdom, and Turkey; the blue blocks mainly include countries such as Germany, Spain, and the Netherlands; and the countries in the yellow and purple regions, such as Switzerland, Sweden, and Japan, are relatively independent and have low degrees of cooperation with the international community, probably because of the relative lagging of their development in this discipline. The thickness of the lines between country nodes is related to the strength of inter-country ties. The results show very strong connections between the United States and countries such as China, Australia, the United Kingdom, and Canada, suggesting that these countries occupy a central position in the field of ML in epilepsy applications.

### 3.5 Distribution by institutions

At present, a total of 1,957 organizations are involved in research on the application of ML in epilepsy. Table 2 shows the top 10 institutions in terms of number of publications. The institution with the highest number of publications is Harvard University (58), followed by the University of London (46). Among the top 10 institutions in terms of the number of publications, four are from the United States, followed by two from China and two from the United Kingdom. It is worth noting that the University of London (0.1), the Chinese Academy of Sciences (0.09), and the University of California (0.07) have relatively high centrality, and a higher mediator centrality of an institution means that the institution has an important role in information transfer (23), which implies that these institutions have an important position in the research of ML in the field of epilepsy applications.

**Table 2:** Top 10 institutions in terms of number of articles issued and the corresponding centrality.

**Rank**	**Institution**	**Publication**	**Centrality**
1	Harvard University	58	0.06
2	University of London	46	0.1
3	University of California System	44	0.07
4	Harvard Medical School	40	0.05
5	University College London	31	0.05
6	University of Melbourne	29	0.06
7	Chinese Academy of Sciences	27	0.09
8	Capital Medical University	27	0.04
9	Massachusetts General Hospital	23	0.01
10	Monash University	23	0.05

**Figure 3:**
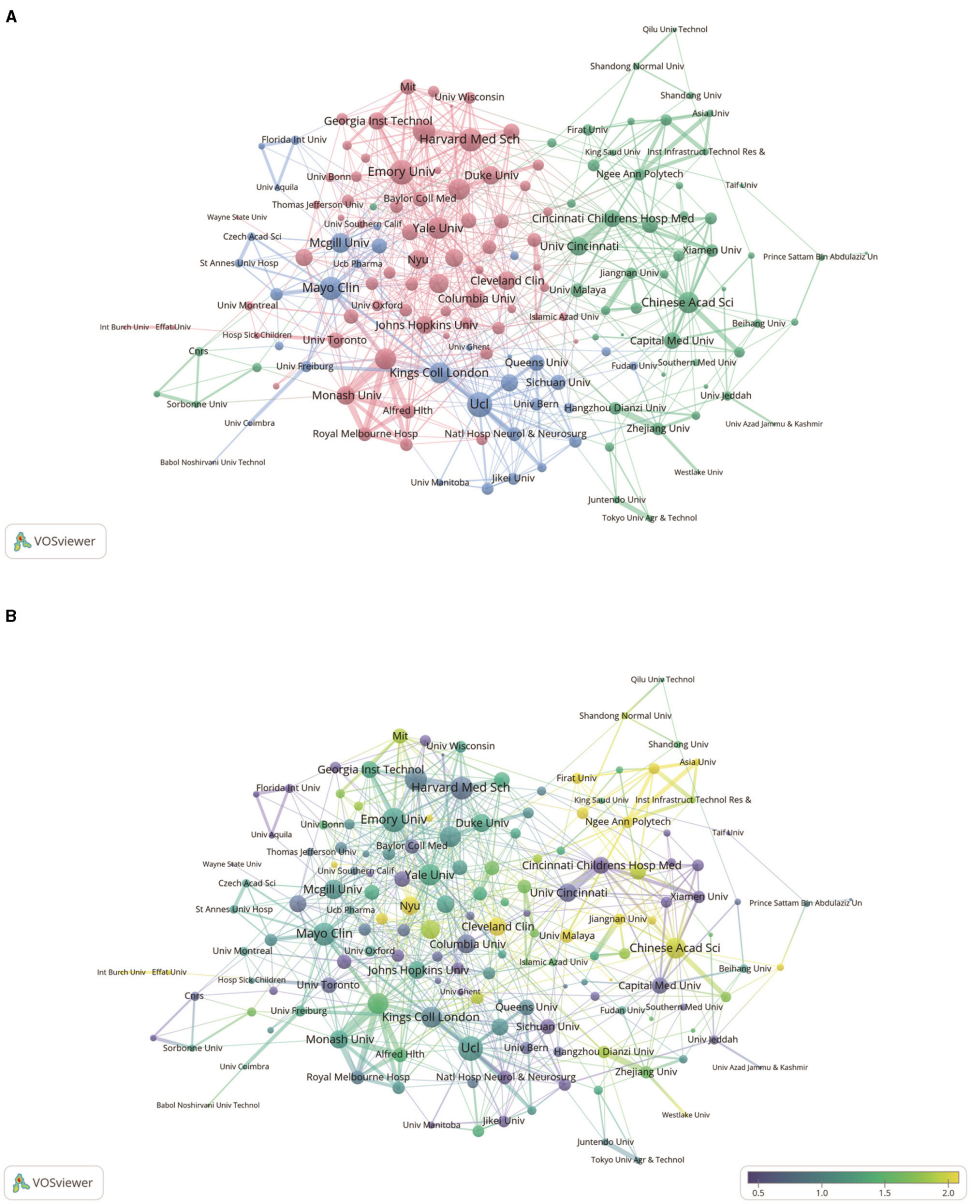
Analysis of network-related institutions. **(A)** Visual analysis of institutional collaboration networks in VOSviewer. Institutions with more than five publications are shown in the figure. Nodes of different colors represent institutions in different clusters, and the size of the nodes indicates their frequency of occurrence. **(B)** Analysis of the number of articles published by institutions in the last 5 years. The heat value of each institution in the last 5 years is obtained by dividing the number of articles published in the last 5 years by the total number of papers.

The purpose of the analysis by research institution was to understand the global distribution of research related to ML in epilepsy and to provide opportunities for collaboration. In VOSviewer, we showed the top 165 institutions with more than five publications and categorized collaborations between institutions into three highly correlated clusters (Figure 3A). Figure 3B shows the ratio of papers published by each institution in the last 5 years to the total number of papers, which is derived by dividing the number of papers published by each institution in the last 5 years that are relevant to the field by the total number of papers published from 2004 to 2023. Figure 3B illustrates the ratio of institutional publications to total publications for the last five years. The purpose of the analysis by research institution was to understand the global distribution of research related to ML in epilepsy and to provide opportunities for collaboration. In VOSviewer, we showed the top 165 institutions with more than five publications and categorized collaborations between institutions into three highly correlated clusters (Figure 3A). Figure 3B shows the ratio of papers published by each institution in the last 5 years to the total number of papers, which is derived by dividing the number of papers published by each institution in the last 5 years that are relevant to the field by the total number of papers published from 2004 to 2023. Figure 3B illustrates the ratio of institutional publications to total publications for the last 5 years. A yellow color indicates a higher percentage, suggesting that these institutions are an emerging force in the field of research. Conversely, if the color leans toward purple, it indicates a lower percentage of published papers, suggesting that these institutions have conducted relatively little research in this area in recent years. According to the display, institutions such as the Chinese Academy of Sciences, New York University and the Cleveland Clinic have seen a significant increase in the amount of research conducted over the past 5 years. In contrast, institutions such as Harvard Medical School, Columbia University, the University of Cincinnati and Cincinnati Children's Hospital have conducted relatively little research in the past five years.

### 3.6 Distribution of authors

The Author Collaboration Network shows the collaborative relationships between different authors, presenting the frequency and closeness of collaboration between authors through graphs and connecting lines (20). Co-citation author analysis refers to two authors whose literature is simultaneously cited by a third author. The higher the co-citation frequency, the closer the collaboration between authors and the stronger the authors' influence (24). By analyzing the number of published papers and authors with the highest co-citation frequency of studies related to the application of ML in epilepsy, we can visualize the authors' influence and research hotspots in the field. Table 3 shows the top 10 authors in terms of number of publications and co-citation frequency. The author with the highest number of publications and co-citation frequency in the field is the same author Acharya, U. Rajendra of Ngee Ann Polytechnic, Singapore (17, 489), which is a good indication of his academic standing in the field of ML and epilepsy. This is followed by Loddenkemper, Tobias of Harvard Medical School, Boston, Massachusetts, USA (13) and Brinkmann, Benjamin H. of Mayo Clinic, Rochester, MN, USA (13), who rank second in terms of number of publications. The second and third most co-cited authors are Andrzejak, Ralph.G of Pompeu Fabra University, Barcelona, Spain (269), Sharma, Manik of Institute of Infrastructure Technology Research and Management, Ahmedabad 380026, India (262).

**Table 3:** The top 10 authors in terms of number of publications and co-citation frequency.

**Rank**	**Author**	**Publications**	**Total link strength**	**Author**	**Co-citations**
1	Acharya, U. Rajendra	17	59	Acharya, U. Rajendra	489
2	Loddenkemper, Tobias	13	91	Andrzejak, Ralph G	269
3	Brinkmann, Benjamin H.	13	136	Sharma, Mona R	262
4	Kuhlmann, Levin	12	97	Subasi, Abdulhamit	257
5	Schulze-Bonhage, Andreas	12	127	Fisher, Robert S	240
6	Duncan, John S.	11	97	Hassan, Ahnaf Rashik	172
7	Westover, M. Brandon	11	113	Mormann, Florian	158
8	Kwan, Patrick	11	106	Bhattacharyya, Anirban	139
9	Dourado, Antonio	10	58	Lihua Guo	136
10	Sharma, Manish	10	28	Gotman, Jean	130

**Figure 4:**
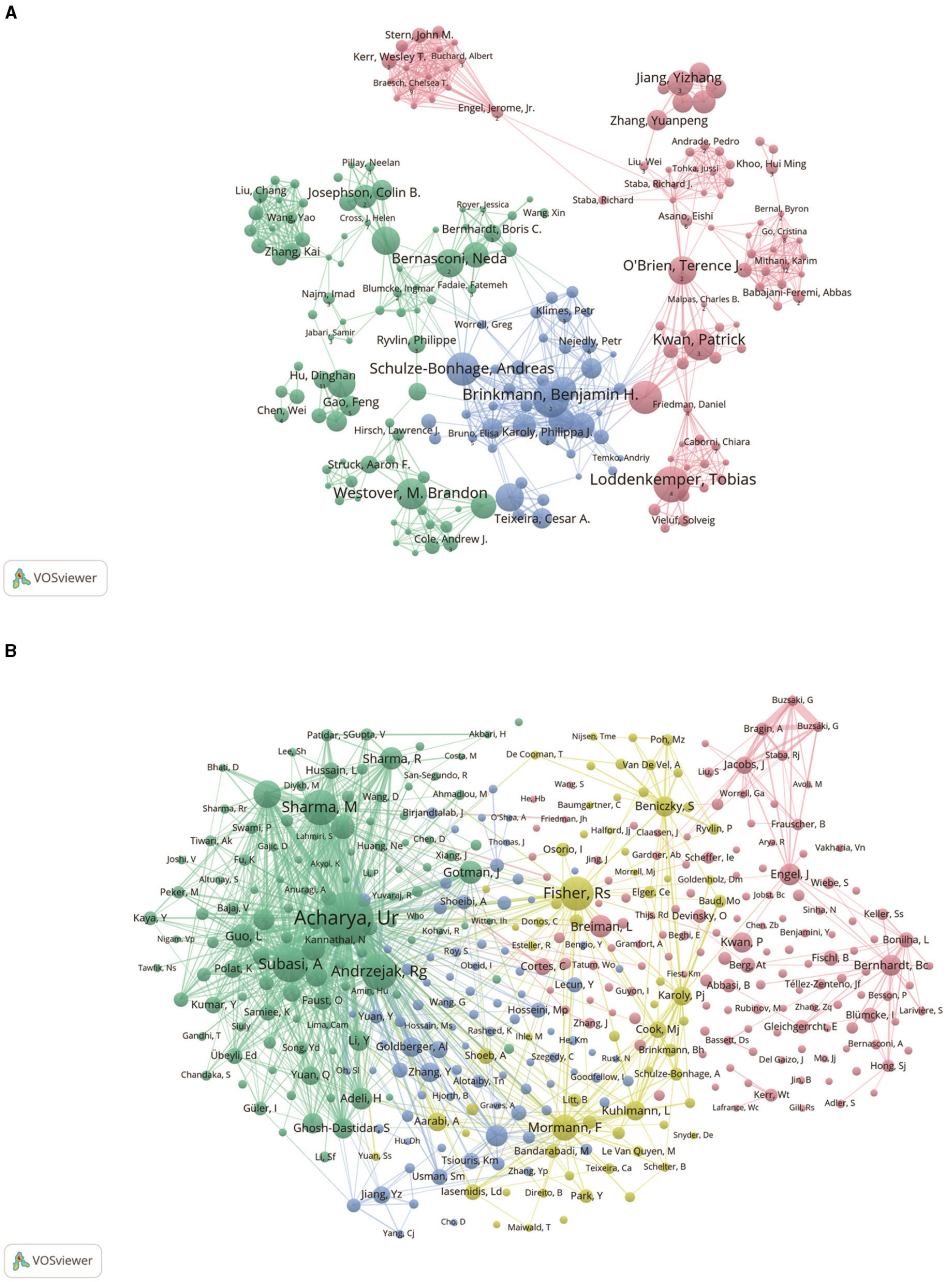
Authorship analysis associated with epilepsy ML research. **(A)** Collaborative networks among authors were observed using VOSviewer. The figure shows the top 300 authors in terms of publications. Nodes of different colors represent authors in different clusters, and the size of the nodes usually represents the number of publications or the frequency of collaboration among authors. **(B)** Authors' co-citation network displayed using VOSviewer. The size of the nodes reflects the number of citations or influence of the authors.

Figure 4A shows the collaboration between authors involved in the study of ML applications in epilepsy. In VOSViewer, authors are divided into three main blocks based on the closeness of collaboration, indicated by different colors. The red block mainly includes authors such as Loddenkemper, Tobias, Kwan, Patrick, etc., the blue block mainly includes authors such as Brinkmann, Benjamin H., Schulaze-Bonhage, Andreas, etc., and the green block mainly includes authors such as Westover, M. Brandon, Bernasconi, Neda and other authors. It shows the collaboration between different authors, which can help researchers better understand the academic collaboration, research hotspots and academic impact in a certain field.

Figure 4B shows the network map of co-cited author relationships. The results show a high degree of consistency in the research focus of the authors of studies related to the application of ML in epilepsy, which are categorized into 4 main blocks. The red block includes authors such as Bernhardt, Bc, Engel, J, the green block includes authors such as Acharya, Ur, Andrzejak, Rg, the yellow block includes authors such as Fisher, Rs, Mormann, F, and the blue block includes authors such as Goldberger, AI, Zhang, Y, and so on.

### 3.7 Distribution of journals

We used the bibliometrics online analysis platform to identify journals with high publication volume and impact in areas related to ML applications in epilepsy. The impact factor (IF) and Journal Citation Report (JCR) quartiles of a journal reflect the impact of the journal (25). JCR partitioning is a process whereby all journals in a given discipline are ranked in descending order of their impact factor for the previous year and then equally divided into four zones, each with an equal share of 25%, Zone 1: top 25% (including 25%); Zone 2: 25%−50% (including 50%).

A total of 372 journals have published articles on this research topic over the past 20 years. The top 10 relevant sources all published at least 21 articles and all were from the USA, UK and Switzerland (Table 4). The most represented journals are “EPILEPSIA,” “BIOMEDICAL SIGNAL PROCESSING AND CONTROL” and “IEEE ACCESS,” with 64, 44, and 43 articles published, respectively.

**Table 4:** Top 10 journals by number of publications, corresponding IF (JCR 2022) and JCR quartile.

**Rank**	**Journal**	**Publications**	**IF (JCR2022)**	**JCR quartile**	**Country**
1	EPILEPSIA	64	5.6	Q1	USA
2	BIOMEDICAL SIGNAL PROCESSING AND CONTROL	44	5.1	Q2	UK
3	IEEE ACCESS	43	3.9	Q2	USA
4	EPILEPSY & BEHAVIOR	35	2.6	Q2	USA
5	FRONTIERS IN NEUROLOGY	32	3.4	Q2	Switzerland
6	FRONTIERS IN NEUROSCIENCE	28	4.3	Q2	Switzerland
7	JOURNAL OF NEURAL ENGINEERING	28	4.0	Q1	UK
8	SENSORS	28	3.9	Q1	Switzerland
9	SCIENTIFIC REPORTS	22	4.6	Q1	UK
10	CLINICAL NEUROPHYSIOLOGY	21	4.7	Q2	UK

**Figure 5:**
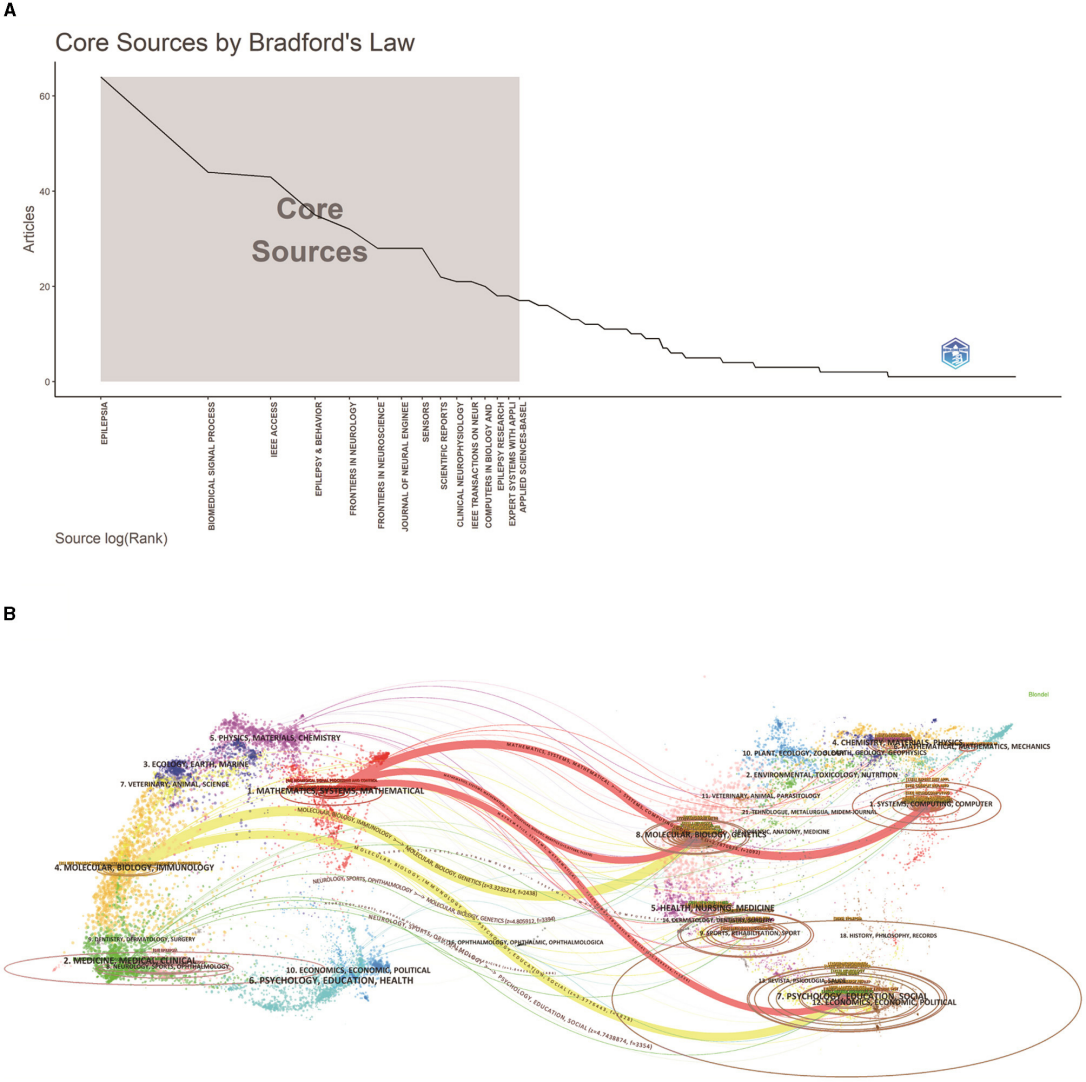
An analysis of journals related to the application of ML in epilepsy. **(A)** A plot of Broadford's Law identifies 15 core journals on ML applications in epilepsy (2004–2023). **(B)** Dual graph overlay of journals. Citing journals are shown on the left, cited journals on the right, and colored paths represent citation relationships.

Journal articles in a discipline are usually concentrated in a certain number of core journals, with the rest dispersed in a large number of related journals in that order (26). Bradford's Law, which describes the distribution of scientific articles across journals, was used to identify 15 core journals that are considered to be preferred by researchers in the field of study (27, 28) (Figure 5A). The number of articles published in core journals in this field is 345, which is 26.9% of the total number of articles.

The journal biplot overlay reflects changes in subject distribution, citation trajectories, and research centers in academic journals (29). As shown in Figure 5B, research on ML applications in epilepsy spans multiple disciplines. The visualized chart is divided into two parts: the left part represents size journals, reflecting the knowledge frontier; the right part represents cited journals, reflecting the knowledge base,and the colored paths indicated the citation relationships (30, 31). In Figure 5B, the thickest bars show the five core citation paths. The topics of Citing Journals are mainly MOLECULAR, BIOLOGY, IMMUNOLOGY, MATHEMATICS, SYSTEMS, MATHEMATICAL namely research frontier. The topics of Cited Journals are mainly MOLECULAR, BIOLOGY, GENETICS, STSTEMS, COMPUTING, COMPUTER, PSYCHOLOGY, EDUCATION, SOCIAL, ECONOMICS, ECONOMIC, POLITICAL, i.e., the knowledge base.

### 3.8 Keyword analysis

As an overview of the core content of the article, keywords can be used to analyze the research frontiers of ML applications in epilepsy. Table 5 shows the top 10 keywords in order of frequency of occurrence. The most frequent keyword was “machine learning” (459), followed by “epilepsy” (387). In addition, “electroencephalogram” (160), “deep learning” (132), and “epilepsy detection” (108) were also keywords that appeared with high frequency, indicating that their corresponding domains were popular in this study.

**Table 5:** Top 10 keywords in terms of frequency of occurrence and the corresponding total link strength.

**Rank**	**Keyword**	**Occurrences**	**Total link strength**
1	Machine learning	459	2,368
2	Epilepsy	387	2,129
3	Eeg	160	865
4	Deep learning	132	742
5	Seizure detection	108	618
6	Feature extraction	94	782
7	Classification	86	466
8	Electroencephalogram	61	338
9	Seizure prediction	55	294
10	Transfer learning	44	486

**Figure 6:**
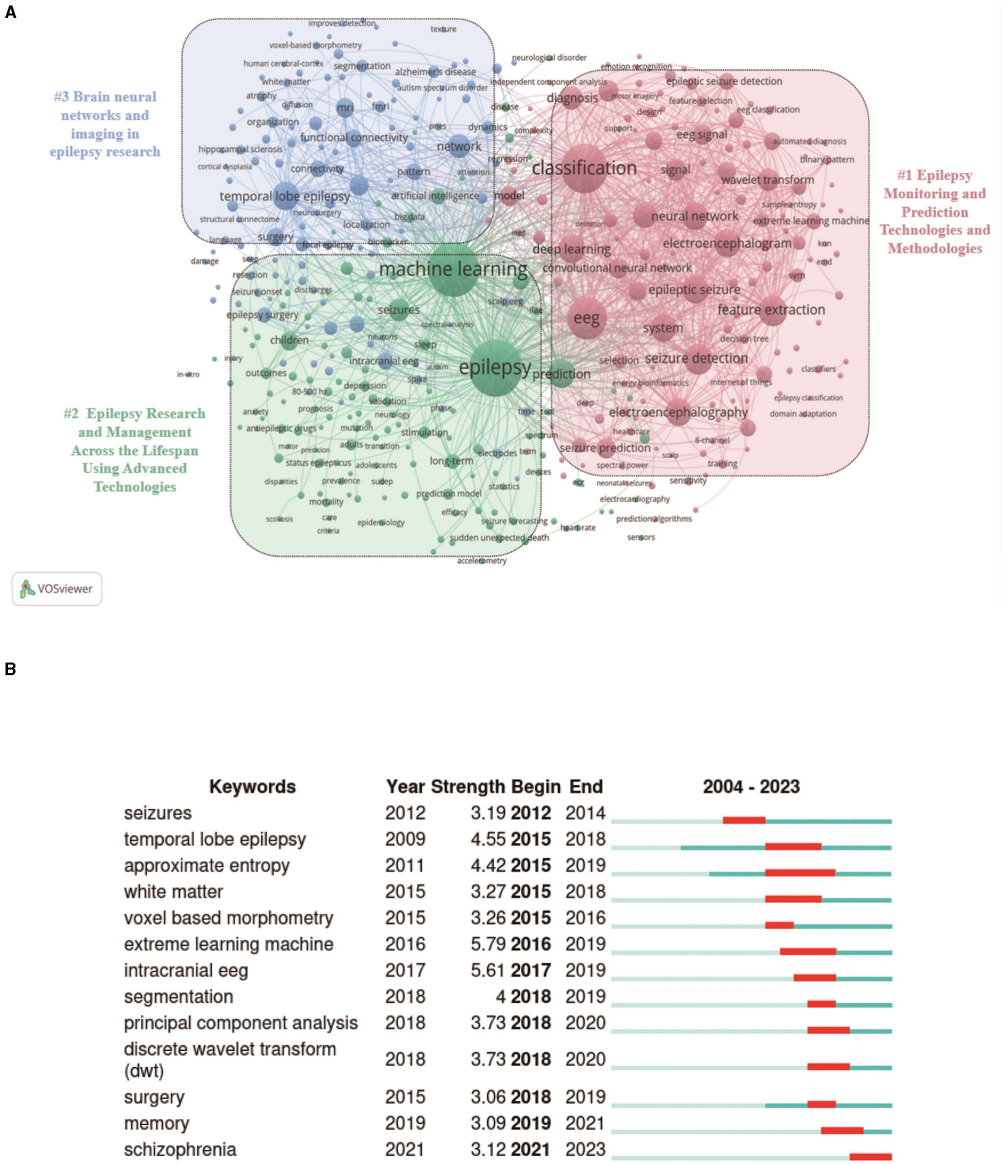
Keyword analysis related to the application of ML in epilepsy. **(A)** Collaborative network visualization of keywords in VOSviewer. Keywords with more than 10 occurrences are shown in the figure. Nodes of different colors represent keywords of different clusters, and the size of the nodes indicates their frequency of occurrence. **(B)** Top 13 keywords with the strongest citation bursts.

The co-occurrence network diagram of the keywords can be seen in VOSviewer (Figure 6A). A total of 448 keywords were screened from the available data, which can be categorized into three groups (Figure 6A). Cluster 1: “Epilepsy monitoring and prediction techniques and methods” in red; Cluster 2: “Research and management of epilepsy across the lifecycle using advanced technologies” in green; and Cluster 3: “Brain neural networks and imaging in epilepsy research” in blue. The size of the keyword circles in each cluster is proportional to their frequency of occurrence. The main terms comprising cluster 1 are “eeg,” “classification,” “seizure detection” and “deep learning” (32–35). All of these terms are closely linked in computer-assisted epilepsy diagnosis and research. Meanwhile, cluster 2 is characterized by the core keywords “epilepsy,” “machine learning,” “seizures” and “prediction” (36, 37), all address important topics in epilepsy research and management. Lastly, cluster 3 is distinguished by “temporal-lobe epilepsy,” “network,” “mri,” “connectivity ” (38–40) as its core keywords. This theme covers several aspects of the field of epilepsy related to neurography, covering relevant techniques, pathologies, mechanistic studies, and clinical applications. At their core, neural networks are computational models inspired by the human brain's structure and function. They consist of layers of nodes, or “neurons,” each capable of performing simple calculations. By processing inputs through these layers, neural networks can recognize patterns, make decisions, and predict outcomes. In epilepsy research, neural networks analyze electroencephalogram (EEG) data to identify patterns that precede seizures, offering a potential for predictive models that could alert patients and physicians to impending epileptic events (41, 42). This technique involves the use of algorithms to examine medical images, such as magnetic resonance imaging (MRI) scans, for the purpose of detecting abnormalities without manual intervention (43). In the context of epilepsy, automatic image analysis can pinpoint areas of the brain affected by epilepsy, known as epileptogenic zones, with remarkable accuracy (44, 45). This capability is particularly valuable for planning surgical interventions, as it helps in precisely identifying the brain regions to be treated or removed. Both neural networks and automatic image analysis exemplify how ML and AI can be harnessed to enhance the accuracy of epilepsy diagnosis and the efficacy of treatment plans. By automating the analysis of complex medical data, these technologies offer a promise of more personalized and predictive healthcare for individuals with epilepsy, moving toward interventions that are tailored to the unique patterns and needs of each patient's condition.

While VOSviewer is effective in showing the co-occurrence status of keywords, it has limitations in illustrating changes in keyword salience (46). The burstiness of a keyword is calculated by weighting the sum of its frequencies in one or more time windows. A keyword is said to be emergent if the probability of these occurrences is above a global threshold that depends on the data (47). The burstiness graph shown in Figure 6B starts in 2012, as this is the first year in which burstiness occurs. To solve this issue, we used CiteSpace software to extract the citation frequency of all keywords, especially the top 13 keywords. The analysis of keyword bursts helps to gain insights into keyword popularity trends and their temporal distribution (24). The most cited keyword is “extreme learning machine.” It is worth noting that the keyword “schizophrenia” has had a higher concentration since 2021, and it is possible that schizophrenia has some correlation with epilepsy.

### 3.9 Highly cited reference analysis

We report the top 10 most co-cited references in Table 6. The Deep convolutional neural network for the automated detection and diagnosis of seizure using EEG signals (41) published by Acharya UR. et al., 2018 in the Computers in Biology and Medicine was the most co-cited paper, with 115 citations in our network and 1,476 citations in the literature. The paper with the second highest number of citations is Truong ND. et al. article on seizure prediction (48) published in NEURAL NETWORKS in 2018, where we have 61 web citations compared to 448 literature citations.

**Table 6:** Top 10 highly cited references.

**Rank**	**Article title**	**Source title**	**Authors**	**Year**	**Cited**	**DOI**
1	Deep convolutional neural network for the automated detection and diagnosis of seizure using EEG signals	COMPUT BIOL MED	Acharya et al.	2018	115	doi: 10.1016/j.compbiomed.2017.09.017
2	Convolutional neural networks for seizure prediction using intracranial and scalp electroencephalogram	NEURAL NETWORKS	Truong et al.	2018	61	doi: 10.1016/j.neunet.2018.04.018
3	Machine learning applications in epilepsy	EPILEPSIA	Abbasi et al.	2019	57	doi: 10.1111/epi.16333
4	An automated system for epilepsy detection using EEG brain signals based on deep learning approach	EXPERT SYST APPL	Ullah et al.	2018	55	doi: 10.1016/j.eswa.2018.04.021
5	Epileptic seizure detection based on EEG signals and CNN	FRONT NEUROINFORM	Zhou et al.	2018	54	doi: 10.3389/fninf.2018.00095
6	Focal onset seizure prediction using convolutional networks	IEEE T BIO-MED ENG	Khan et al.	2018	52	doi: 10.1109/TBME.2017.2785401
7	Performance evaluation of empirical mode decomposition, discrete wavelet transform, and wavelet packed decomposition for automated epileptic seizure detection and prediction	BIOMED SIGNAL PROCES	Alickovic et al.	2018	51	doi: 10.1016/j.bspc.2017.07.022
8	A long short-term memory deep learning network for the prediction of epileptic seizures using EEG signals	COMPUT BIOL MED	Tsiouris et al.	2018	51	doi: 10.1016/j.compbiomed.2018.05.019
9	Efficient epileptic seizure prediction based on deep learning	IEEE T BIOMED CIRC S	Daoud et al.	2019	50	doi: 10.1109/TBCAS.2019.2929053
10	Seizure prediction—ready for a new era	NAT REV NEUROL	Kuhlmann et al.	2018	47	doi: 10.1038/s41582-018-0055-2

**Figure 7:**
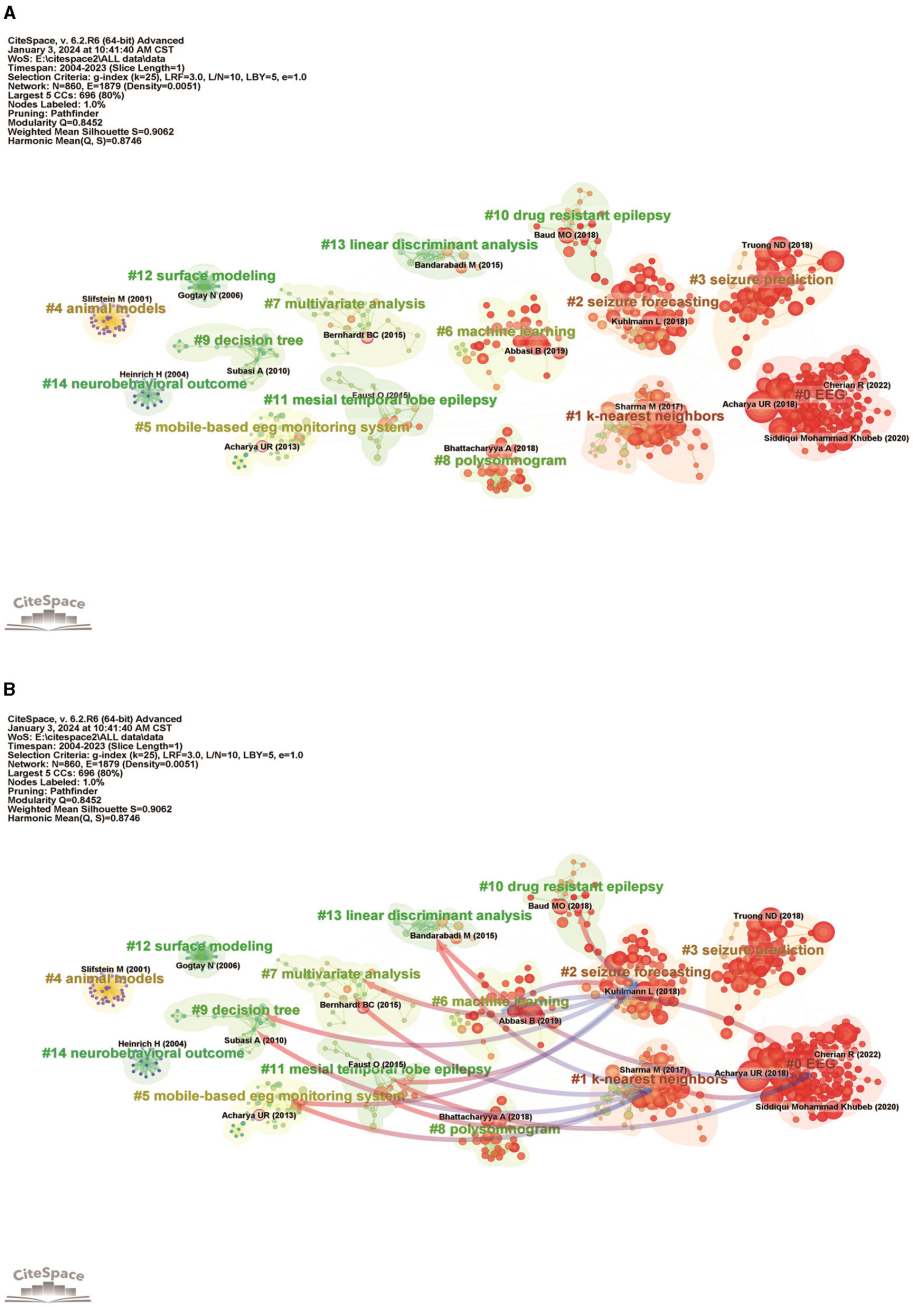
Analysis of references related to the application of ML in epilepsy. **(A)** Cluster map analysis of references via CiteSpace. A total of 14 clusters were obtained. Different color blocks represent different reference clusters. **(B)** Cluster dependencies (clusters with blue starting point clusters depend on clusters with red arrows).

We performed cluster analysis and cluster dependency analysis of cited literature using CiteSpace 6.2.R6 advanced visualization software. In bibliometric analysis, tracing the sources of reference clustering can indicate the research base and research hotspots in academic fields (49). As shown in Figure 7A, the thematic clustering map of the study area identified 14 clusters with significant modularity and silhouette scores (*Q* = 0.8452; *S* = 0.9062) (50). These two metrics are critical for assessing the effectiveness of a graphical visualization (51). A *Q*-value >0.3 and an *S*-value >0.5 indicate that the clustering structure is stable and highly persuasive. The largest cluster was labeled “EEG” (cluster #0), followed by clusters focused on “k nearest neighbors” (cluster #1), “seizure prediction” (cluster #2), and “seizure prediction” (cluster #3). Other important clusters focus on “Mobile-based EEG monitoring,” “Machine learning,” “Multivariate analysis,” “Decision trees” and “Temporal lobe mesial epilepsy.” It is worth noting that the top ranked item by centrality is Bernhardt (52) in Cluster #7, with centrality of 0.12. The second one is Acharya (53) in Cluster #5, with centrality of 0.11.

Dependencies between clusters are based on certain clusters, and there will always be some clusters that provide a knowledge base for other clusters. As shown in Figure 7B, “mobile-based eeg monitoring system” (#5), “multivariate analysis” (#7) with red arrows, “mesial temporal lobe epilepsy” (#11), and “linear discriminant analysis” (#13) clusters are the ones with blue starting ends of the arrows “k-nearest neighbors” (#1) clustering is the knowledge base for #1 clustering cites the literature on #5, #7, #11, and #13 clustering, #1 clustering evolved from these clusters and is at the forefront of research. “EEG” (#0) clustering and “seizure forecasting” (#2) clustering are the basis for “machine learning” (#6) clustering. However, it also relies on “k-nearest neighbors” (#1), “mesial temporal lobe epilepsy” (#11), “linear discriminant analysis” (#12), “linearized temporal lobe epilepsy” (#13), and “linear discriminant analysis” (#14).

## 4 Discussion

### 4.1 General distribution

The application of ML in epilepsy is an emerging trend of research in the field of neurology and many studies have yielded promising results. Seizure Prediction Models: The number of studies in the field using deep learning has increased as new efficient models have been proposed, culminating in a model that can predict seizures minutes before they occur, with significantly improved accuracy (54). This predictive ability is critical for patients to take preemptive measures to ensure safety and mitigate the effects of seizures. Automatic detection of epileptiform discharges: Another significant contribution was the development of an ML algorithm capable of automatically detecting epileptiform discharges in EEG recordings (55). Traditionally, this task requires the expertise of a trained neurologist, but the ML model has shown considerable accuracy, thereby simplifying the diagnostic process and enabling faster patient evaluation. MRI Image Analysis for Surgical Planning: In a groundbreaking study, researchers applied convolutional neural networks (CNNs) to analyze MRI scans of epilepsy patients (56). The model successfully identified epileptogenic zones with high precision, aiding neurosurgeons in planning surgical interventions with improved accuracy, potentially increasing the success rate of epilepsy surgeries. Drug Response Prediction: Utilizing ML to predict patient response to anti-epileptic drugs (AEDs) represents another area of success. By analyzing patient data, including genetic information, seizure types, and treatment history, ML models have been able to predict with significant accuracy which patients are likely to respond well to specific AEDs (57), paving the way for personalized treatment strategies. Automated Video Analysis for Seizure Detection: An innovative application of ML in epilepsy involves the use of video analysis to detect physical signs of seizures, particularly in monitoring situations such as long-term EEG video telemetry (58). By training algorithms on video data, researchers have developed systems that can accurately identify seizure-related movements, reducing the reliance on manual monitoring and improving patient safety. These examples underscore the diverse and impactful ways in which ML is revolutionizing the field of epilepsy treatment and research. Through enhancing diagnostic accuracy, predicting seizures, aiding in surgical planning, and personalizing treatment approaches, ML is at the forefront of efforts to improve the quality of life for individuals living with epilepsy.

Our study aimed to offer new insights for future research in machine learning applications within epilepsy studies. In this study, we analyzed 1,284 articles in the Web of science Core Collection database from January 1, 2004, to December 22, 2023, regarding the use of ML in epilepsy. The incremental increase in the number of articles suggests indicates a growing interest in the application of ML in epilepsy. The average number of citations per article is 20.14, showing the impact and relevance of this research area. International collaboration exists but there is room for strengthening, especially in fostering more global partnerships beyond the major contributions of the United States and China. Through CiteSpace, VOSviewer and Bibliometrix R software, the literature was comprehensively analyzed by combining country/region, institution, authors, keywords and references to explore in depth the knowledge structure, research hotspots and development trends in the field, to determine the accuracy of ML algorithms in epilepsy research, and to explore its great potential. It provides a basis for the subsequent development and accurate identification of epilepsy management programs. We delve into the reasons behind our findings by analyzing the methodologies employed, the nature of the datasets used, and the current technological advancements in ML. Our analysis reveals that the success of ML in epilepsy research is partly due to the increasing accuracy and sophistication of algorithms in handling complex datasets, reflecting broader trends in AI.

According to an analysis of annual publication and citation trends (shown in Figure 1B), the latter decade has seen a significant increase in publications and citations, reflecting the broader integration of AI and ML with medical research. This surge highlights the potential of AI to revolutionize epilepsy research and treatment, with a peak in 2022 underscoring the rapid growth of the field. However, a slight decline in 2023 due to delays in the publication of papers emphasizes the need for ongoing monitoring to fully understand developments in the field.

As shown in Figure 4, we analyze the research co-authorship networks through bibliometrics, and we can determine the structure and intensity of international collaborations in the epilepsy research community. This analysis can highlight prolific institutions, countries, and researchers driving innovation in ML applications for epilepsy. In Figures 5, 6, bibliometric studies can track the evolution of research topics in epilepsy ML applications through keyword and topic trend analysis. This analysis reveals how international collaborations contribute to the diversification of research agendas, the emergence of new research directions, and the adoption of cutting-edge methods. Collaboration facilitates the cross-fertilization of ideas, often resulting in innovative approaches that address long-standing challenges in the diagnosis and treatment of epilepsy. In the citation analysis in Figure 7, the bibliometric tool demonstrates the impact of the cited literature, an impact that underscores the value of collaborative efforts in generating broadly resonant research findings that advance the field's collective understanding and application of ML in epilepsy.

As shown in Tables 1, 2, and Figure 2, among the 87 countries/territories that published literature on ML applications in epilepsy, the United States demonstrated the largest number of publications, citation frequency, and proportion of international collaborations, showing its academic status in this research field. In addition, China and India, which ranked second and third in terms of number of publications and citation frequency, also showed rapid growth. In terms of the number of publications, four of the top 10 institutions are from the United States, two from China and two from the United Kingdom. The UK's University of London Center degree is the highest of all institutions, indicating its dominant position in global collaboration in this field. In addition to this, countries such as China, India, Australia and Canada are also widely involved in research and collaboration on ML and epilepsy.

As can be seen from Tables 3, 6 and Figure 4B, Acharya, U. Rajendra is ranked first in terms of number of publications and co-citation frequency and is far ahead of the other authors, which indicates his outstanding impact in the field related to the application of ML in epilepsy. However, yet the strength of linkage is weak in inter-author collaborations and collaboration with other researchers is more lacking. It is worth mentioning that the article “Deep Convolutional Neural Networks for Automatic Detection and Diagnosis of Epileptic Seizures Using Electroencephalographic Signals” by Acharya, U. Rajendra et al. in Computers in Biology and Medicine in 2018 is the most frequently cited article in the field. Integration of the above data analyzes that Acharya, U. Rajendra has had a significant impact on this field of research.

The analysis of Figure 5B shows that in the past nearly two decades, the main disciplines at the forefront of ML in epilepsy as cross-disciplinary research have generally ranged from “molecular/biological/immunological/mathematical/systems” to “molecular/biological/genetic/computer/psychological/educational/ social/economic/political.” Such a change implies a subtle shift in the research themes of the field, from a focus on the use of antiepileptic drugs and genetic mechanisms in epileptic populations to the prediction, prevention and management of seizures. These shifts highlight the research hotspots and trends in the field.

### 4.2 Hotspots and frontiers

The keyword analysis helps to understand the frontiers and hot topics in the research field of ML in epilepsy. The main keywords of existing studies include “machine learning,” “epilepsy,” “EEG,” “deep learning,” “epilepsy detection,” “feature extraction,” and “classification” (Table 5), which are mainly related to epileptic seizure prediction. For instance, the focus on “seizure detection” reflects the urgent need for real-time monitoring solutions. This trend is indicative of a broader shift toward leveraging ML to solve practical healthcare challenges. The co-occurrence network Figure 6A shows that high-frequency keywords present several popular research directions in previous studies, including epilepsy prediction technology, epilepsy management, and epilepsy imaging histology. It is worth noting that the keyword burst Figure 6B shows that the appearance of “schizophrenia” may have some correlation with epilepsy, but excessive extrapolation or assumption of correlation should be avoided when using ML. Correlation studies of these two disorders need to be confirmed by more in-depth clinical and basic science studies to improve the understanding and treatment of these two disorders.

In summary, this study analyzes the literature published on the topics of “Machine Learning” and “Epilepsy” over the last two decades, primarily using bibliometric methods, and identifies the countries, institutions, authors, and journals that have made significant contributions to the field during this period. Bibliometric analysis offers a powerful lens through which to understand the dynamics and impact of international and national collaborations in the realm of ML applications for epilepsy. These collaborations are pivotal in driving forward the boundaries of knowledge, fostering innovation, and ensuring that advancements in the field are widely accessible and impactful. Through detailed bibliometric scrutiny, stakeholders can gain valuable insights into effective collaboration models, promising research directions, and strategies to enhance the global impact of their work. Currently, research in this area focuses on feature extraction using various ML algorithms to explore seizure prediction, epilepsy classification, and epilepsy neuroimaging. Despite the impressive growth, challenges remain, particularly in enhancing international collaboration and diversifying the research focus to include emerging trends and interdisciplinary approaches. ML applied to epilepsy is a new field, and future research trends will focus on better integration of clinical data and epilepsy neuroimaging to develop more accurate ML algorithms and apply these algorithms to clinical settings. The popularity of certain topics, such as seizure detection and management, indicates a growing demand for solutions that can be directly applied to patient care. The emphasis on pharmacologic and surgical treatment prognosis using ML points to a shift toward personalized medicine, where treatments are tailored based on predictive analytics. The alignment between technological advancements in ML and the evolving needs of epilepsy research signifies a synergistic relationship. This connection is fueled by the mutual goal of improving patient outcomes and the quality of life for those living with epilepsy, highlighting the role of ML in facilitating significant breakthroughs in the field. We believe that with the combined efforts of researchers, ML is expected to provide neurologists with better tools and rationale to more accurately provide personalized treatment and comprehensive management plans for patients with epilepsy.

## 5 Limitations

This study is the first to use bibliometric visualization to analyze studies related to the application of ML in epilepsy in the last 20 years. First of all, this study only included literature from the WOSCC database, excluding data from other databases (e.g., PubMed, Scopus, and Google Scholar, etc.). Second, only English literature was included, and papers in other languages failed to be included. Third, new papers published after the search date could not be included in this study because the database was kept open. Therefore, there may be some degree of missing and biased data. However, the concept of Science Citation Index is based on Bradford's law in bibliometrics, which can be used to define a core set of journals or publications, and journals included in the Science Citation Index Extended (SCI-E) in the Web of Science database are referred to as world leading journals because of their rigorous selection process (59). Therefore, publications included in WOS can represent the level of research in the field.

## Data availability statement

The original contributions presented in the study are included in the article/supplementary material, further inquiries can be directed to the corresponding author.

## Author contributions

QH: Data curation, Formal analysis, Software, Validation, Visualization, Writing – original draft, Writing – review & editing. XL: Conceptualization, Formal analysis, Methodology, Project administration, Supervision, Validation, Writing – original draft, Writing – review & editing. Z-CX: Project administration, Supervision, Validation, Writing – review & editing. X-YY: Formal analysis, Validation, Writing – review & editing.

## References

[1] Fisher RS Acevedo C Arzimanoglou A Bogacz A Cross JH Elger CE . ILAE official report: a practical clinical definition of epilepsy. Epilepsia. (2014) 55:475–82. 10.1111/epi.12550 24730690

[2] Scheffer IE Berkovic S Capovilla G Connolly MB French J Guilhoto L . ILAE classification of the epilepsies position paper of the ILAE commission for classification and terminology. Epilepsia. (2017) 58:512–21. 10.1111/epi.13709 28276062 PMC5386840

[3] Fodjo JNS Makoy YL Colebunders R. Epilepsy prevention. Lancet Lond Engl. (2019) 394:2072. 10.1016/S0140-6736(19)31906-3 31818410

[4] Guekht A Brodie M Secco M Li S Volkers N Wiebe S. The road to a World Health Organization global action plan on epilepsy and other neurological disorders. Epilepsia. (2021) 62:1057–63. 10.1111/epi.16856 33675058

[5] Cendes F McDonald CR. Artificial intelligence applications in the imaging of epilepsy and its comorbidities: present and future. Epilepsy Curr. (2022) 22:91–6. 10.1177/15357597211068600 35444507 PMC8988724

[6] Karakis I. Sage against the machine: promise and challenge of artificial intelligence in epilepsy. Epilepsy Curr. (2022) 22:279–81. 10.1177/15357597221105139 36285200 PMC9549233

[7] Beam AL Kohane IS. Big data and machine learning in health care. JAMA. (2018) 319:1317–8. 10.1001/jama.2017.18391 29532063

[8] Deo RC. Machine learning in medicine. Circulation. (2015) 132:1920. 10.1161/CIRCULATIONAHA.115.001593 26572668 PMC5831252

[9] Kerr WT McFarlane KN. Machine learning and artificial intelligence applications to epilepsy: a review for the practicing epileptologist. Curr Neurol Neurosci Rep. (2023) 23:869–79. 10.1007/s11910-023-01318-7 38060133

[10] Jordan MI Mitchell TM. Machine learning: trends, perspectives, and prospects. Science. (2015) 349:255–60. 10.1126/science.aaa8415 26185243

[11] Abbasi B Goldenholz DM. Machine learning applications in epilepsy. Epilepsia. (2019) 60:2037. 10.1111/epi.16333 31478577 PMC9897263

[12] Lee HM Fadaie F Gill R Caldairou B Sziklas V Crane J . Decomposing MRI phenotypic heterogeneity in epilepsy: a step towards personalized classification. Brain. (2021) 145:897–908. 10.1093/brain/awab425 34849619 PMC9050524

[13] Caciagli L Bassett DS. Epilepsy imaging meets machine learning: a new era of individualized patient care. Brain. (2022) 145:807–10. 10.1093/brain/awac027 35307732

[14] Hicks D Wouters P Waltman L de Rijcke S Rafols I. Bibliometrics: the Leiden Manifesto for research metrics. Nature. (2015) 520:429–31. 10.1038/520429a 25903611

[15] Wan Y Shen J Ouyang J Dong P Hong Y Liang L . Bibliometric and visual analysis of neutrophil extracellular traps from 2004 to 2022. Front Immunol. (2022) 13:1025861. 10.3389/fimmu.2022.1025861 36341351 PMC9634160

[16] Guo Y Xu Z-Y-R Cai M-T Gong W-X Shen C-H. Epilepsy with suicide: a bibliometrics study and visualization analysis via CiteSpace. Front Neurol. (2022) 12:823474. 10.3389/fneur.2021.823474 35111131 PMC8802777

[17] Liu C Zhou W Mao Z Li X Meng Q Fan R . Bibliometric analysis of ferroptosis in acute kidney injury from 2014 to 2022. Int Urol Nephrol. (2023) 55:1509–21. 10.1007/s11255-022-03456-2 36611104

[18] Zhang D Zhu W Guo J Chen W Gu X. Application of artificial intelligence in glioma researches: a bibliometric analysis. Front Oncol. (2022) 12:978427. 10.3389/fonc.2022.978427 36033537 PMC9403784

[19] Chen C. CiteSpace II: detecting and visualizing emerging trends and transient patterns in scientific literature. J Am Soc Inf Sci Technol. (2006) 57:359–77. 10.1002/asi.20317

[20] van Eck NJ Waltman L. Software survey: VOSviewer, a computer program for bibliometric mapping. Scientometrics. (2010) 84:523–38. 10.1007/s11192-009-0146-3 20585380 PMC2883932

[21] Aria M Cuccurullo C. Bibliometrix: an R-tool for comprehensive science mapping analysis. J Informetr. (2017) 11:959–75. 10.1016/j.joi.2017.08.007

[22] Durieux V Gevenois PA. Bibliometric indicators: quality measurements of scientific publication. Radiology. (2010) 255:342–51. 10.1148/radiol.09090626 20413749

[23] Brandes U. A faster algorithm for betweenness centrality^*^. J Math Sociol. (2001) 25:163–77. 10.1080/0022250X.2001.9990249

[24] Yuan W-C Zhang J-X Chen H-B Yuan Y Zhuang Y-P Zhou H-L . A bibliometric and visual analysis of cancer-associated fibroblasts. Front Immunol. (2023) 14:1323115. 10.3389/fimmu.2023.1323115 38173726 PMC10762783

[25] Dai N Li J Ren L Bu Z. Gender representation on editorial boards of leading oncology journals. ESMO Open. (2022) 7:100590. 10.1016/j.esmoop.2022.100590 36174363 PMC9588884

[26] Brookes BC. “Sources of information on specific subjects” by S.C. Bradford. J Inf Sci. (1985) 10:173–5. 10.1177/016555158501000406

[27] Ablakimova N Smagulova GA Rachina S Mussina AZ Zare A Mussin NM . Bibliometric analysis of global research output on antimicrobial resistance among pneumonia pathogens (2013–2023). Antibiotics. (2023) 12:1411. 10.3390/antibiotics12091411 37760709 PMC10525339

[28] Huang R Zhang M Lu Y Xu D Liu Y Jin M . Effects of intestinal microbes on rheumatic diseases: a bibliometric analysis. Front Microbiol. (2023) 13:1074003. 10.3389/fmicb.2022.1074003 36699603 PMC9870327

[29] Chen C Leydesdorff L. Patterns of connections and movements in dual-map overlays: a new method of publication portfolio analysis. J Assoc Inf Sci Technol. (2014) 65:334–51. 10.1002/asi.22968

[30] Wan Y Shen J Hong Y Liu J Shi T Cai J. Mapping knowledge landscapes and emerging trends of the biomarkers in melanoma: a bibliometric analysis from 2004 to 2022. Front Oncol. (2023) 13:1181164. 10.3389/fonc.2023.1181164 37427124 PMC10327294

[31] Zhang J Song L Xu L Fan Y Wang T Tian W . Knowledge domain and emerging trends in ferroptosis research: a bibliometric and knowledge-map analysis. Front Oncol. (2021) 11:686726. 10.3389/fonc.2021.686726 34150654 PMC8209495

[32] Caro V Ho J-H Witting S Tobar F. Modeling neonatal EEG using multi-output gaussian processes. IEEE ACCESS. (2022) 10:32912–27. 10.1109/ACCESS.2022.3159653

[33] Zhang Q Ding J Kong W Liu Y Wang Q Jiang T. Epilepsy prediction through optimized multidimensional sample entropy and Bi-LSTM. Biomed Signal Process Control. (2021) 64:102293. 10.1016/j.bspc.2020.102293

[34] Yang Y Nhan DT Eshraghian JK Maher C Nikpour A Kavehei O . Multimodal AI system for out-of-distribution generalization of seizure identification. IEEE J Biomed Health Inform. (2022) 26:3529–38. 10.1109/JBHI.2022.3157877 35263265

[35] Golmohammadi M Torbati AHHN de Diego SL Obeid I Picone J. Automatic analysis of EEGs using big data and hybrid deep learning architectures. Front Hum Neurosci. (2019) 13:76. 10.3389/fnhum.2019.00076 30914936 PMC6423064

[36] Bruno E Simblett S Lang A Biondi A Odoi C Schulze-Bonhage A . Wearable technology in epilepsy: the views of patients, caregivers, and healthcare professionals. Epilepsy Behav. (2018) 85:141–9. 10.1016/j.yebeh.2018.05.044 29940377

[37] Attia TP Crepeau D Kremen V Nasseri M Guragain H Steele SW . Epilepsy personal assistant device-a mobile platform for brain state, dense behavioral and physiology tracking and controlling adaptive stimulation. Front Neurol. (2021) 12:704170. 10.3389/fneur.2021.704170 34393981 PMC8358117

[38] Lee HW Arora J Papademetris X Tokoglu F Negishi M Scheinost D . Altered functional connectivity in seizure onset zones revealed by fMRI intrinsic connectivity. Neurology. (2014) 83:2269–77. 10.1212/WNL.0000000000001068 25391304 PMC4277677

[39] Vaessen MJ Jansen JFA Vlooswijk MCG Hofman PAM Majoie HJM Aldenkamp AP . White matter network abnormalities are associated with cognitive decline in chronic epilepsy. Cereb Cortex. (2012) 22:2139–47. 10.1093/cercor/bhr298 22038907

[40] Caciagli L Wandschneider B Xiao F Vollmar C Centeno M Vos SB . Abnormal hippocampal structure and function in juvenile myoclonic epilepsy and unaffected siblings. Brain. (2019) 142:2670–87. 10.1093/brain/awz215 31365054 PMC6776114

[41] Acharya UR Oh SL Hagiwara Y Tan JH Adeli H. Deep convolutional neural network for the automated detection and diagnosis of seizure using EEG signals. Comput Biol Med. (2018) 100:270–8. 10.1016/j.compbiomed.2017.09.017 28974302

[42] Rasheed K Qayyum A Qadir J Sivathamboo S Kwan P Kuhlmann L . Machine learning for predicting epileptic seizures using EEG signals: a review. IEEE Rev Biomed Eng. (2021) 14:139–55. 10.1109/RBME.2020.3008792 32746369

[43] Chen W Wang Y Ren Y Jiang H Du G Zhang J . An automated detection of epileptic seizures EEG using CNN classifier based on feature fusion with high accuracy. BMC Med Inform Decis Mak. (2023) 23:96. 10.1186/s12911-023-02180-w 37217878 PMC10201805

[44] Lopes MA Goodfellow M Terry JR. A model-based assessment of the seizure onset zone predictive power to inform the epileptogenic zone. Front Comput Neurosci. (2019) 13:25. 10.3389/fncom.2019.00025 31105545 PMC6498870

[45] Coito A Plomp G Genetti M Abela E Wiest R Seeck M . Dynamic directed interictal connectivity in left and right temporal lobe epilepsy. Epilepsia. (2015) 56:207–17. 10.1111/epi.12904 25599821

[46] Zhiguo F Ji W Shenyuan C Guoyou Z Chen K Hui Q . A swift expanding trend of extracellular vesicles in spinal cord injury research: a bibliometric analysis. J Nanobiotechnology. (2023) 21:289. 10.1186/s12951-023-02051-6 37612689 PMC10463993

[47] Kim MC Nam S Wang F Zhu Y. Mapping scientific landscapes in UMLS research: a scientometric review. J Am Med Inform Assoc JAMIA. (2020) 27:1612–24. 10.1093/jamia/ocaa107 33059367 PMC7647344

[48] Truong ND Nguyen AD Kuhlmann L Bonyadi MR Yang J Ippolito S . Convolutional neural networks for seizure prediction using intracranial and scalp electroencephalogram. Neural Netw. (2018) 105:104–11. 10.1016/j.neunet.2018.04.018 29793128

[49] Song B Lin Z Feng C Zhao X Teng W. Global research landscape and trends of papillary thyroid cancer therapy: a bibliometric analysis. Front Endocrinol. (2023) 14:1252389. 10.3389/fendo.2023.1252389 37795362 PMC10546338

[50] Sabé M Chen C El-Hage W Leroy A Vaiva G Monari S . Half a century of research on posttraumatic stress disorder: a scientometric analysis. Curr Neuropharmacol. (2024) 22:736–48. 10.2174/1570159X22666230927143106 37888890 PMC10845098

[51] Tao S Tang X Yu L Li L Zhang G Zhang L . Prognosis of coronary heart disease after percutaneous coronary intervention: a bibliometric analysis over the period 2004–2022. Eur J Med Res. (2023) 28:311. 10.1186/s40001-023-01220-5 37658418 PMC10472664

[52] Bernhardt BC Hong S-J Bernasconi A Bernasconi N. Magnetic resonance imaging pattern learning in temporal lobe epilepsy: classification and prognostics. Ann Neurol. (2015) 77:436–46. 10.1002/ana.24341 25546153

[53] Acharya UR Vinitha Sree S Swapna G Martis RJ Suri JS. Automated EEG analysis of epilepsy: a review. Knowl-Based Syst. (2013) 45:147–65. 10.1016/j.knosys.2013.02.014

[54] Shoeibi A Khodatars M Ghassemi N Jafari M Moridian P Alizadehsani R . Epileptic seizures detection using deep learning techniques: a review. Int J Environ Res Public Health. (2021) 18:5780. 10.3390/ijerph18115780 34072232 PMC8199071

[55] Jing J Sun H Kim JA Herlopian A Karakis I Ng M . Development of expert-level automated detection of epileptiform discharges during electroencephalogram interpretation. JAMA Neurol. (2020) 77:103–8. 10.1001/jamaneurol.2019.3485 31633740 PMC6806668

[56] Kaestner E Rao J Chang AJ Wang ZI Busch RM Keller SS . Convolutional neural network algorithm to determine lateralization of seizure onset in patients with epilepsy. Neurology. (2023) 101:e324–35. 10.1212/WNL.0000000000207411 37202160 PMC10382265

[57] Corrales-Hernández MG Villarroel-Hagemann SK Mendoza-Rodelo IE Palacios-Sánchez L Gaviria-Carrillo M Buitrago-Ricaurte N . Development of antiepileptic drugs throughout history: from serendipity to artificial intelligence. Biomedicines. (2023) 11:1632. 10.3390/biomedicines11061632 37371727 PMC10295412

[58] Yang Y Sarkis RA Atrache RE Loddenkemper T Meisel C. Video-based detection of generalized tonic-clonic seizures using deep learning. IEEE J Biomed Health Inform. (2021) 25:2997–3008. 10.1109/JBHI.2021.3049649 33406048

[59] Zhu J Liu W. A tale of two databases: the use of Web of Science and Scopus in academic papers. Scientometrics. (2020) 123:321–35. 10.1007/s11192-020-03387-8

